# A tough egg to crack: recreational boats as vectors for invasive goby eggs and transdisciplinary management approaches

**DOI:** 10.1002/ece3.1892

**Published:** 2016-01-11

**Authors:** Philipp E. Hirsch, Irene Adrian‐Kalchhauser, Sylvie Flämig, Anouk N'Guyen, Rico Defila, Antonietta Di Giulio, Patricia Burkhardt‐Holm

**Affiliations:** ^1^ Research Centre for Sustainable Energy and Water SupplyBaselSwitzerland; ^2^ Program Man‐Society‐EnvironmentUniversity of BaselBaselSwitzerland; ^3^ Department of Biological SciencesUniversity of AlbertaEdmontonABCanada

**Keywords:** Attachment strength, biological invasions, conservation management, desiccation tolerance, *Neogobius melanostomus*, *Ponticola kessleri*, saltatorial dispersal

## Abstract

Non‐native invasive species are a major threat to biodiversity, especially in freshwater ecosystems. Freshwater ecosystems are naturally rather isolated from one another. Nonetheless, invasive species often spread rapidly across water sheds. This spread is to a large extent realized by human activities that provide vectors. For example, recreational boats can carry invasive species propagules as “aquatic hitch‐hikers” within and across water sheds. We used invasive gobies in Switzerland as a case study to test the plausibility that recreational boats can serve as vectors for invasive fish and that fish eggs can serve as propagules. We found that the peak season of boat movements across Switzerland and the goby spawning season overlap temporally. It is thus plausible that goby eggs attached to boats, anchors, or gear may be transported across watersheds. In experimental trials, we found that goby eggs show resistance to physical removal (90 mN attachment strength of individual eggs) and stay attached if exposed to rapid water flow (2.8 m·s^−1^for 1 h). When exposing the eggs to air, we found that hatching success remained high (>95%) even after eggs had been out of water for up to 24 h. It is thus plausible that eggs survive pick up, within‐water and overland transport by boats. We complemented the experimental plausibility tests with a survey on how decision makers from inside and outside academia rate the feasibility of managing recreational boats as vectors. We found consensus that an installation of a preventive boat vector management is considered an effective and urgent measure. This study advances our understanding of the potential of recreational boats to serve as vectors for invasive vertebrate species and demonstrates that preventive management of recreational boats is considered feasible by relevant decision makers inside and outside academia.

## Introduction

Naturally, individuals of any purely aquatic species cannot move freely between water bodies because of the dendritic nature of watersheds and due to the isolation of catchments from one another (Thienemann 1950). It is therefore intriguing that aquatic ecosystems are disproportionally impacted by rapid range expansions of invasive species across watersheds (Rahel 2007). The most probable reasons behind this large‐scale spread of invasive species are human activities. Humans break down natural barriers to dispersal in aquatic ecosystems by, for example, building shipping ways that connect major catchments (Rahel 2007). For example, the Rhine–Main–Danube channel provides a link between two major European watersheds. Humans also provide vectors which realize the uptake of propagules in one system, the translocation, and the release into another system (Johnson et al. 2001). For example, many bivalves are able to adhere to aquatic equipment and survive exposure to air during transport (Johnson et al. 2001; Clarke Murray et al. 2011). Whereas the loss of natural barriers is hard to reverse, the management of human vectors is possible. Hence, if we want to prevent the negative impacts of invasive species on native aquatic ecosystems, we have to manage human vectors (Hirsch et al. 2015; N'Guyen et al. 2015). Importantly, such a management should follow the precautionary principle. As illustrated by the proverb that an ounce of prevention is better than a pound of cure, a preventive management is the most cost‐efficient strategy against the negative impacts of invasive species (Leung et al. 2002). In the case of an imminent invasion, acting timely is essential.

To be effective and successful, the management of invasive species' vectors needs to be installed as soon as an invasion is anticipated, and it needs to fulfill two prerequisites. Firstly, it needs to rest on empirical knowledge on how plausible certain propagules and certain vectors are for the invasion process (Johnson et al. 2001). Secondly, management measures have to be feasible in light of both scientific knowledge and of perceived barriers to its implementation (Tzankova and Concilio 2015). The feasibility of a measure cannot be established by scientific knowledge alone (Gozlan et al. 2013). Human vectors need to be managed by humans, and perceptions of people outside academia, including stakeholders, ultimately determine whether a measure is implemented (Gozlan et al. 2013; Tzankova and Concilio 2015).

In this study, we test for both the plausibility and the management feasibility of a vector. Our study species are invasive Ponto‐Caspian gobies (round goby *Neogobius melanostomus* and bighead goby *Ponticola kessleri*). They are likely to cause economic and ecological harm and have recently established a potential source population in the river Rhine in Switzerland (Hirsch et al. 2015; N'Guyen et al. 2015). From this localized population, further invasions into previously goby‐free Swiss waters can be expected (Kornis et al. 2012; Kalchhauser et al. 2013). Gobies are small benthic fish that are incapable of prolonged swimming. The invasion of gobies follows a saltatorial pattern: Instead of continuously expanding along a watercourse, new populations establish rapidly in isolated water bodies and areas far away from the presumed source population (Kalchhauser et al. 2013). This suggests that their dispersal is aided by some vector. Commercial ship traffic, transporting propagules in ballast water tanks, is assumed to be a major long‐distance vector for gobies, although empirical evidence for the plausibility of this vector is still lacking. Spread into isolated and smaller water bodies is more likely to be realized by recreational rather than commercial boat traffic (Johnson et al. 2001; Poos et al. 2010). In the High Rhine, natural upstream dispersal of gobies is unlikely because of weirs and dams which pose effective in‐stream barriers for similar‐sized native benthic fish species (Hirsch et al. 2015; N'Guyen et al. 2015). Isolated alpine lakes in Switzerland are not connected to the Rhine via navigable waterways. However, recreational boats are frequently transported overland between lakes and also across in‐stream barriers. Consequently, overland transport by recreational boats is a possible vector that requires further study. The introduction of invasive gobies in Switzerland provides a suitable case in point to empirically explore if recreational boats are plausible as a vector and how feasible a preventive management of this vector is perceived inside and outside academia.

**Figure 1:**
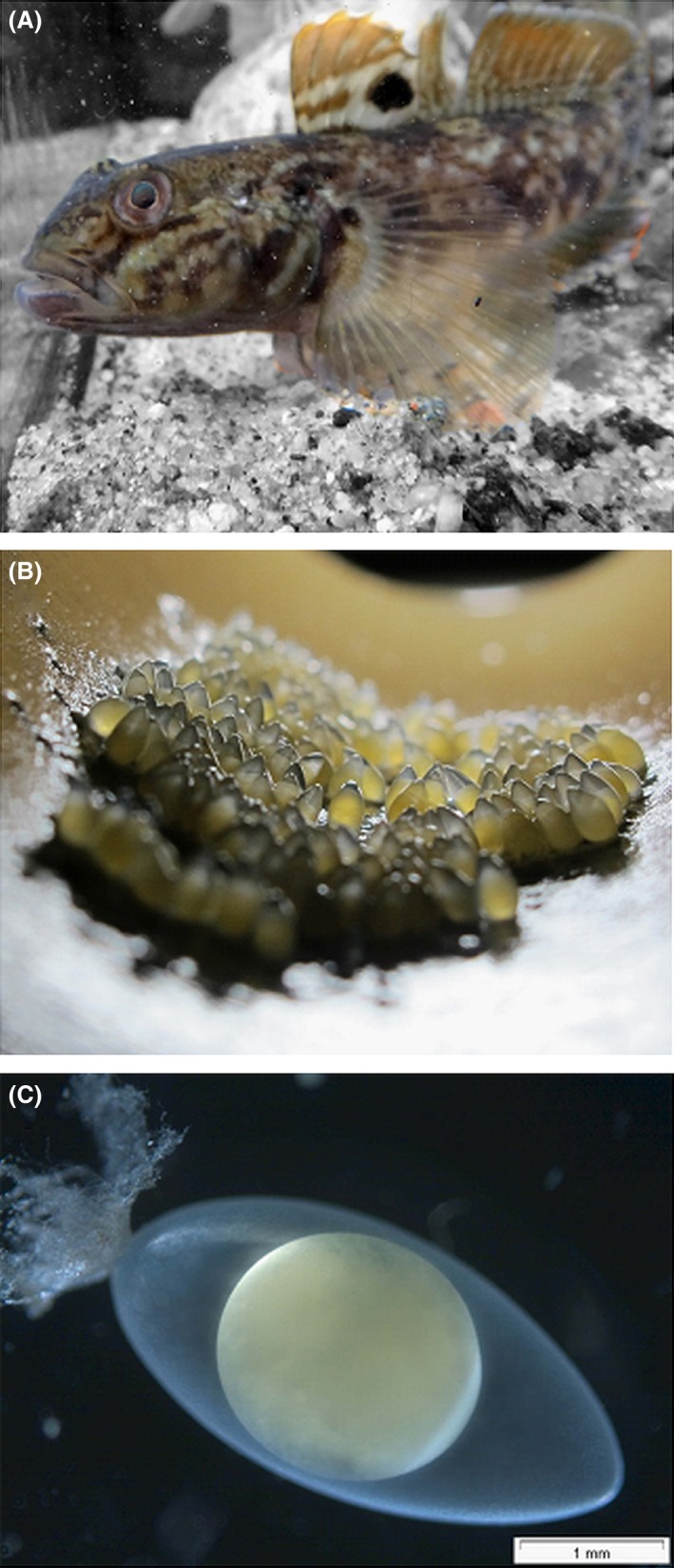
Invasive goby and adhesive eggs as possible propagules. (A) Round goby (*Neogobius melanostomus*) in an aquarium. (B) Adhesive eggs spawned into a PVC pipe as part of a spawning trap (see Appendix S4 for more details). (C) Microscopic picture of an egg showing the attaching filaments (scale bar = 1 mm).

From an invasion science point of view, the precautionary principle makes the preventive management of any vector imperative as soon as the vector's plausibility is established (Leung et al. 2002). The plausibility of a vector is given when vectors and propagules occur at the same time in the same place (hence allowing for pick up of propagules by vectors), and when propagules survive pick up, transport and release (Drake and Mandrak 2014). Eggs are one of the most frequently mentioned, yet previously unexplored, propagules of invasive fish. For invertebrate species, resting stages and eggs are well‐described as propagules (Havel and Shurin 2004). However, despite much speculation in the literature about fish eggs as potential propagules, this assumption remains to be empirically tested (see Appendix S1 for a list of references that mention eggs as invasive goby propagules). In the case of invasive gobies, speculations on egg transport rely on the observation that gobies readily spawn adhesive eggs onto artificial substrates (Fig. 1, Appendix S2). Anecdotal reports suggest that goby eggs are laid onto boat hulls or gear such as anchors, and it has thus been speculated that goby eggs may stay attached to these substrates when the boats travel in water or are transported overland (Appendix S1).

In this study, we empirically address the plausibility of recreational boats as vectors and goby eggs as propagules as well as the feasibility of management based on the following questions: (1) Do vector and propagules temporally overlap in activity to make a pick up and translocation plausible? (2) Are propagules able to survive conditions during a translocation? (3) How do relevant decision makers value a preventive management of the vector? To address question (1), we examine the temporal overlap of vector movements and propagule availability using data on local boat movements and on the local goby spawning season. We consider a large temporal overlap as a necessary condition for the vector to pick up propagules. To address question (2), we experimentally test the ability of eggs to survive relevant transport conditions. We postulate (i) that eggs need to be capable of resisting drag forces as they are moved by a vector within or between water bodies, attached to boats or gear, and (ii) that eggs need to be tolerant to desiccation as they are moved from one catchment to the other. To address question (3), we asked relevant experts inside and outside academia how efficient, difficult or urgent they would rate a preventive management of recreational boats as vectors. We argue that the combination of empirically confirmed plausibility of a specific vector and the consensus on the need for its preventive management would allow to more specifically counteract the human spread of invasive gobies (cf. N'Guyen et al. 2015).

## Materials and Methods

### Question (1)

To establish the plausibility of eggs serving as propagules and boats as vectors, we explored the temporal overlap of vector activity and the propagule availability. Data on recreational boat movements in Switzerland between 2009 and 2013 were obtained from a survey on boats in Switzerland carried out at the Swiss Federal Institute of Aquatic Science and Technology (EAWAG; see Weissert 2013 for details). The data (kindly provided by L. DeVentura [EAWAG]) were further analyzed to explore how many boats are moved overland during which time of the season. This analysis resulted in a subset of 684 recorded overland transports that we used in this study to demonstrate how these were distributed across one season. To investigate the temporal overlap of boat movement across in‐stream barriers, we compiled data for boat passages across the dam Birsfelden which is upstream of the source population (for information on the questionnaire and data on all boat passages across all in‐stream barriers upstream of the source population in the Rhine, see Appendix S3).

Goby eggs were retrieved from the recently detected source population in the local harbor at Basel, Switzerland (47.587518°N, 7.593447°E), with specifically designed spawning traps consisting of clay pots and PVC pipes as artificial spawning substrates (see Appendix S4 for details). Clutches found in the traps were transported in a bucket of aerated harbor water and photographed upon arrival in the laboratory. Digital photographs were used to count the number of eggs. Each clutch was kept in a separate 10 L overflow tank supplied by 14.8°C (±0.1°C) UV‐treated tap water.

### Question (2)

To experimentally test the propagules' endurance of relevant transport conditions, we measured (i) the attachment strength and (ii) the desiccation resistance of goby eggs. To measure attachment strength in the laboratory, the clutches attached to their artificial substrate (PVC pipes) were fixed to a glass dish and peak resistance force was recorded for each individual egg pulled perpendicular from the substrate using tweezers (force gauge: Model M7i, Mark‐10 Corporation, Copiague, NY, USA, sensor Mark‐10 via Plug TestTMTechnology). Outliers (0.1% of all data) caused by handling errors (such as tweezers slipping) were identified using the Grubb's outliers test and removed from the data set.

To measure egg attachment under flow conditions, eggs attached to artificial substrates from the spawning traps were exposed to water flow in a swim tunnel (185 L, 50 Hz, ^®^Loligo Systems, Tjele, Denmark). Clutches were attached in the tunnel so that flow would hit the eggs on their longitudinal side at a right angle. The tested velocity of 2.8 m·s^−1^ corresponds to approximated velocities occurring on the hull of a recreational boat traveling with 10 km·h^−1^ upstream the Rhine. Egg attachment under flow conditions was expressed as number of eggs remaining attached after 1 h of water flow exposure. We assumed that a boat with the above‐mentioned cruising speed would need 1 h to travel from the harbor where gobies have established upstream to the next major in‐stream barrier.

To test for survival of eggs under air exposure, four different exposure periods (0.25, 0.5, 12 h or 24 h) were applied with one half of a clutch exposed to air in an incubator (Model IPP 300, ^®^Memmert, Schwabach, Germany) and the other half remaining in the tank as a control. The incubator temperature was identical to the mean summer air temperature during spawning season (incubator: 18°C, field: 18°C). The incubating humidity was also similar to field conditions (incubator: 60–85% (median 80%), field 75% (median), all field data from Federal Office of Meteorology and Climatology MeteoSwiss, 2013). After exposure, the clutch halves from the incubator were placed into tanks until hatching started. The hatching rate can be different among different clutches. To account for this variation, we set the hatching rate of the untreated half of each clutch as the standard successful hatching rate for each clutch and the hatching rate in the treated half was expressed in % of this untreated clutch‐specific “standard hatching rate.” Hatching success was calculated as number of hatched embryos divided by the number of viable eggs for each clutch half.

### Question (3)

A successful installation of preventive measures against invasive species requires the cooperation and compliance of relevant decision makers inside and outside academia. To explore the feasibility of a preventive management of recreational boats, we developed a questionnaire completed by participants of a transdisciplinary workshop which we organized (see N'Guyen et al. (2015) for background information on our transdisciplinary approach). The workshop's participants were certified experts (holding academic degrees in relevant subjects or holding professional positions in relevant areas; example: researchers, environmental authorities) and noncertified experts (expertise built on experience; example: representatives of local fishing clubs) (Defila and Di Giulio 2015). All experts were decision makers representing different groups which we classified as representatives of civil society 1 (public and private companies, *n* = 6), civil society 2 (angler associations and NGOs, *n* = 4), authorities and administration (*n* = 7), or scholars (*n* = 4). All participants received an individual handout together with the questionnaire. The handout described a check‐clean‐dry routine as a possible management measure to prevent recreational boats acting as vectors for invasive gobies (Appendix S5). Participants were then asked to rate effectiveness, urgency, and perceived difficulty of implementation by making crosses on a linear scale which we then recorded as numerical values by overlaying a scale from 1 to 10, with 0.1 intervals. The scale reached from very urgent, effective, and difficult to not urgent, effective or difficult (see Appendix S6 for the actual questions).

## Results

### Question (1)

**Figure 2:**
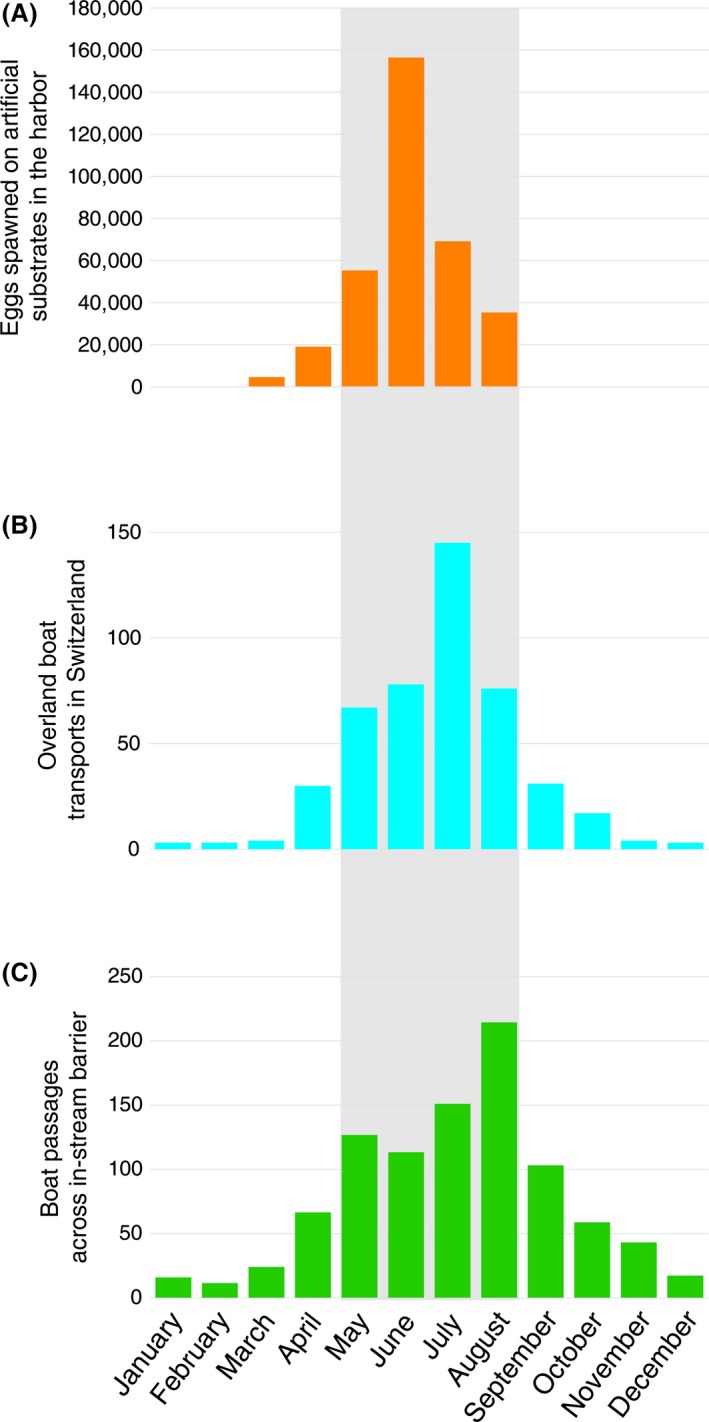
Goby spawning season overlaps with vector activity. (A) Number of goby eggs spawned on artificial substrates by the potential source population in 2013. (B) Number of overland recreational boat transports between 2009 and 2013 (data kindly provided by L. DeVentura). (C) Number of boat passages across the in‐stream barrier adjacent to the source population (watergate Birsfelden) between 2009 and 2013 (see Appendix S5 for all in‐stream barriers upstream of the source population).

During the spawning season, we could retrieve an estimated 350,000 goby eggs by providing and regularly clearing artificial spawning substrates in the local harbor (Fig. 2A). Testing for the temporal overlap between vector activity and propagule availability, we found that overland boat transfers and passages across in‐stream barriers overlapped with the peak of the local goby spawning season (Fig. 2B and C).

### Question (2)

**Figure 3:**
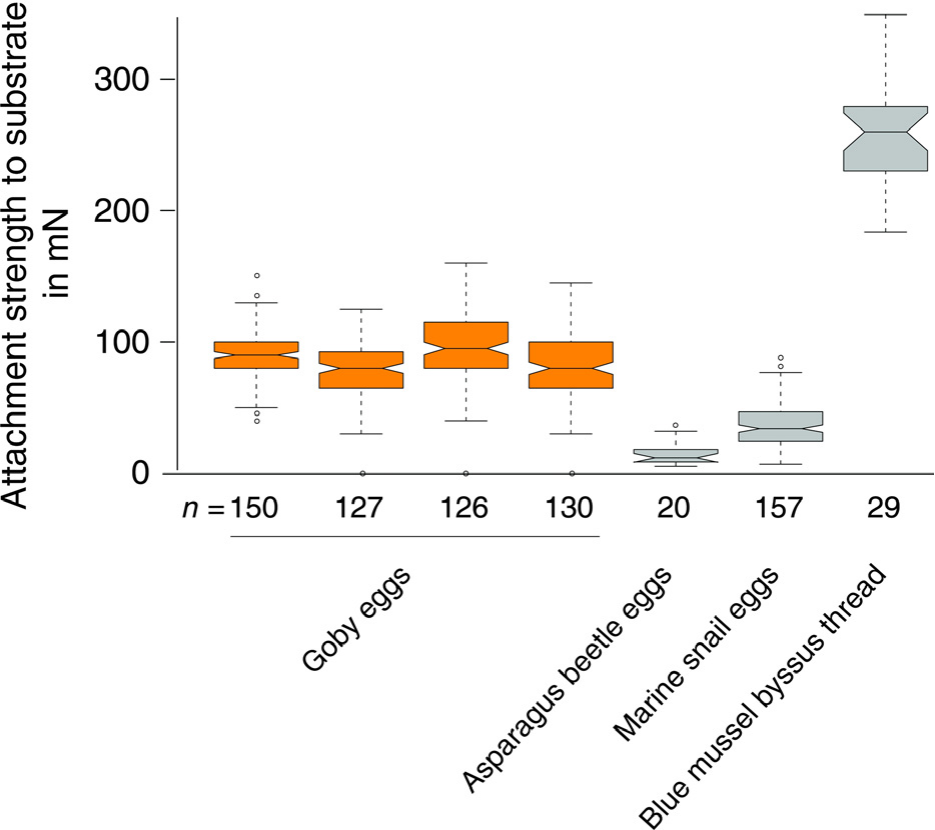
Force measurements reveal attachment strength of goby eggs. Data show peak resistance to perpendicular pulling force in mN. For illustration, the published attachment strengths of asparagus beetle eggs (*Crioceris asparagi*) (Voigt and Gorb 2010), marine snail eggs (*Melanochlamys diomedea*) (Castro and Podolsky 2012), and blue mussel byssus threads (*Mytilus edulis*) (Brenner and Buck 2010) are shown. Nongoby data were extracted from figures in the respective articles using the software GetDataGraphDigitizer v. 2.26 (www.getdata-graph-digitizer.com).

**Figure 4:**
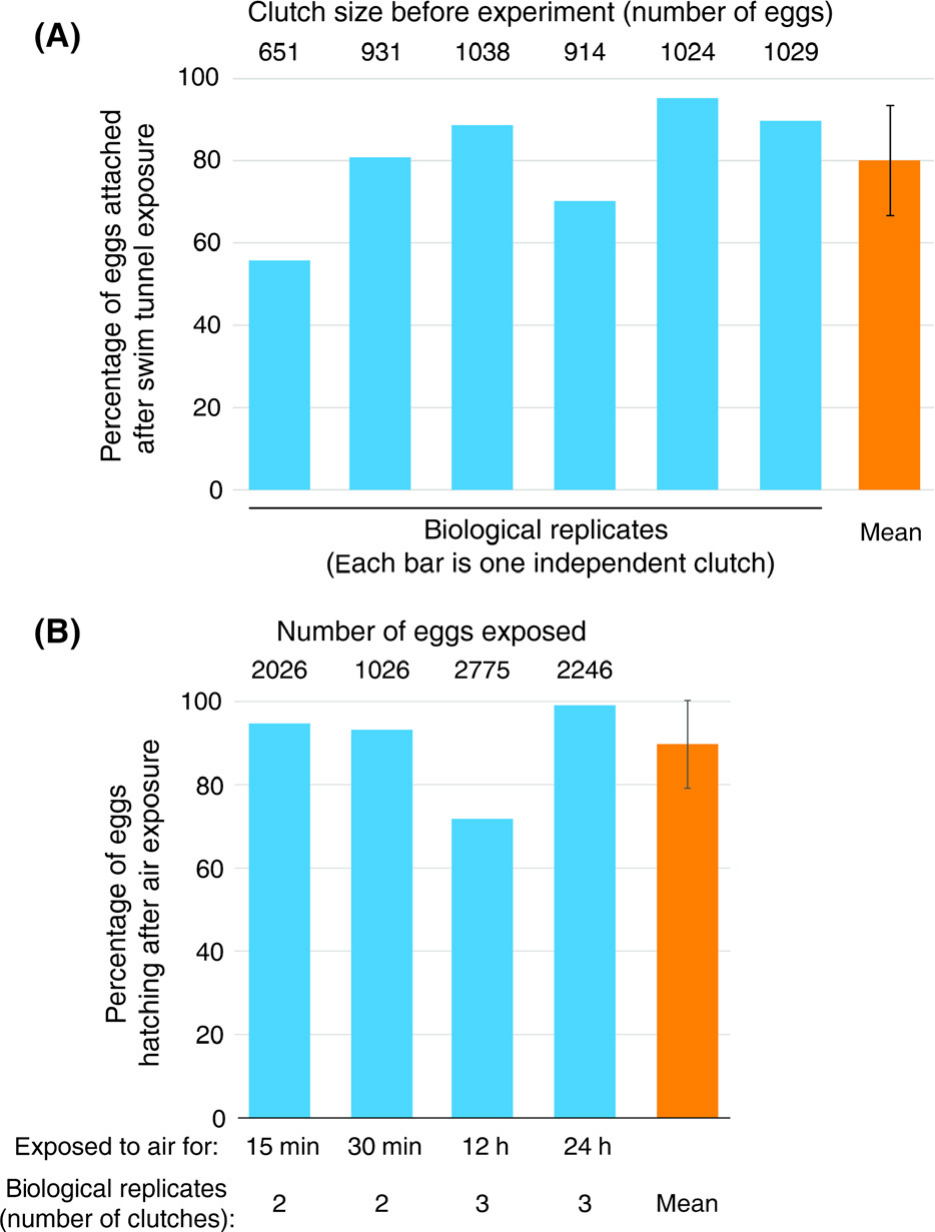
Eggs remain attached in water flow and air exposure does not affect hatching success. (A) Data show attachment in % of remaining eggs after exposure to a water flow of 2.8 m·s^−1^ for 1 h in a swim tunnel. (B) Data show hatching success relative to untreated control. Untreated controls consisted of eggs from the same clutch that remained in water. Error bars denote standard deviation.

(i) Using peak force measurements, we found that the force required to remove a naturally spawned individual egg from an artificial surface is 90 mN (±8.04 standard deviation = SD; Fig. 3). We further explored how naturally spawned eggs would adhere to the substrate under simulated field conditions in a swim tunnel and found that after one hour of simulated boat travel, on average 80% (±13.04 SD) of all eggs remained attached (Fig. 4A). (ii) To test the plausibility that eggs taken out of the water survive as propagules, we investigated egg survival under air exposure and found that eggs exposed to air for 0.15–24 h had a mean hatching rate of 94% (±12.16 SD), with all clutches synchronously hatching after air exposure (Fig. 4B, Appendix S3).

### Question (3)

**Figure 5:**
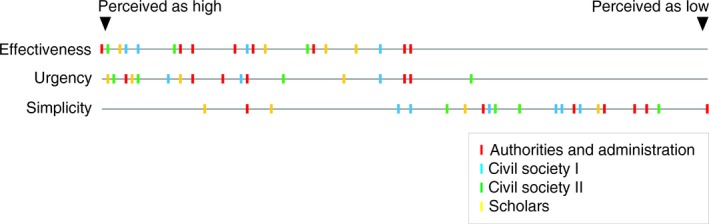
Certified and non‐certified experts from inside and outside academia perceive vector management as effective and urgent, but see barriers to its implementation (ease of implementation is termed “simplicity”). Civil Society 1 (*n* = 6): public and private companies, civil society 2 (*n* = 4): angler associations and NGOs concerned with nature conservation, authorities and administration (*n* = 7): for example, county board, scholars (*n* = 4): scientists interested in invasive species.

When decision makers were asked to rate a “check–clean–dry” measure as a preventive management tool, all but one respondents found the measure urgent and effective (Fig. 5). However, the decision makers also saw barriers to the potential implementation of the measure (Fig. 5, Appendix S8).

## Discussion

Our results confirm the plausibility of recreational boats as vectors of goby eggs. Answering question (1), we could demonstrate that recreational boats are moved in high numbers during the spawning season and that gobies spawn eggs onto artificial substrates. Answering question (2), we found our experiments to reveal resistance of goby eggs to (i) physical forces and (ii) air exposure. Overall, our results provide the first empirical test for eggs attached to boats or gear as plausible propagules of invasive fish. Answering question (3), we learned that decision makers, consisting of certified and noncertified experts, consider a preventive management urgent and effective, albeit with some barriers to implementation.

### Empirical evidence suggests the plausibility of recreational boats as vectors and eggs as propagules

The overlap between vector activity and propagule availability makes pick up of eggs by boats plausible. This is especially relevant since harbors and marinas have previously been found to be primary invasion hot spots of gobies and other aquatic invasive species (Kalchhauser et al. 2013). The frequent establishment of invasive goby populations in harbors and the concentration of recreational boat traffic in marinas make both a temporal and spatial co‐occurrence of vectors and propagules likely (Clarke Murray et al. 2011; Drake and Mandrak 2014). Our results confirm this co‐occurrence empirically. The high attachment strength of eggs on artificial surfaces suggests that the propagules can remain attached to the vector during within‐water transport. The high strength of goby egg attachment becomes evident from comparing the attachment forces of goby eggs with other species' eggs or attachment organs that serve the purpose of resisting drag forces to increase survival. For example, the attachment forces of marine snail eggs *Melanochlamys diomedea* that withstand tidal and wave forces in marine systems are on average lower than those observed in goby eggs. The swim tunnel results demonstrate under more realistic conditions that the observed attachment strength of goby eggs is indeed high enough to prevent goby eggs from being washed from the surface of boats, anchors, or gear when transported within water.

The survival of goby eggs during air exposure fulfills an important prerequisite for eggs to serve as plausible propagules on boats: The propagules are able to withstand conditions during overland transport. The high survival of fish eggs even after air exposure was unexpected: Why would fish eggs survive out of the very element they evolved in? In fact, the survival of anamniotic amphibian eggs in air has been previously acknowledged, and a recent review suggests that the ability to survive in air might also be an underappreciated ability in fish eggs (Martin and Carter 2013). For example, some mudskipper species' eggs develop out of water in an excavated air chamber (Ishimatsu et al. 2009). The resistance to air exposure in both mudskipper and goby eggs makes sense in an evolutionary context. These two fish genera are closely related, and egg adhesion is believed to be a characteristic trait within the taxonomic group of Gobioidei, of which many representatives spawn in tidal zones (Thacker 2009). Adhesive eggs that are resistant to air exposure would be conceivable to evolve as an adaptation to such habitats. In cyprinid fish species, exposure to air has been found to desynchronize hatching (Fisk et al. 2013). In our experiments, however, both control and air‐exposed eggs showed synchronized hatching. This indicates that the development of goby eggs appeared to be unaffected by the desiccation treatment. Invasive goby larvae rapidly start feeding externally and show a survival of over 95% in the 3 months posthatching (Bonislavskaya et al. 2014). This might further increase their chances to survive if released into a new environment. In summary, adhesive fish eggs that can survive within‐water transport and air exposure might have previously underestimated capabilities to serve as propagules. Importantly, our work supports the common notion in invasion biology that a single translocation event might well suffice to establish a population if enough propagules (i.e., eggs) hatch and survive upon arrival in a new environment (Sakai et al. 2001).

### Caveats on the experimental design and interpretation of results

The experiments were designed to test for the plausibility of eggs as propagules. The flow resistance and desiccation experiments were conducted under conditions that were as close to reality as possible. Drag forces in the swim tunnel approximated drag forces acting upon the eggs if they are attached to a boat cruising upstream. The conditions applied for the desiccation test were chosen to represent realistic but replicable field conditions. Naturally, other factors such as wind exposure could influence humidity and temperature and hence egg survival. However our tests were not designed to explore which kind of factors would affect hatching rates to which degree. We aimed at investigating vector plausibility through empirical tests of whether goby eggs can at all survive such conditions and thus are able serve as propagules for a translocation.

The role of human vectors in the dispersal of freshwater vertebrate species is still poorly understood although correlative data clearly hints at human factors playing a substantial role in, for example, fish invasions (Leprieur et al. 2008). For round goby in the Great Lakes, genetic data suggests a role for commercial ships as vectors. The more cargo traffic between distant harbors, the more closely are the harbor populations related to each other, suggesting an exchange of individuals between harbors realized by ships (LaRue et al. 2011). Recent reviews on aquatic invertebrate propagules increasingly acknowledge the need for more empirical studies complementing the correlative knowledge created by genetic studies (Incagnone et al. 2015). The notion that anamniotic eggs can serve as propagules for freshwater fish has long been resting on circumstantial evidence and anecdotal reports (Appendix S1). Only a few early works have explored the plausibility of fish eggs as propagules (Preusse 1924; Schiemenz 1925; but see Oulton et al. (2013) for a recent example). Our study is the first to address this question for invasive fish.

However, it is much more important to consider the need for action that is evident from the precautionary principle than to lament the past or current level of scientific evidence (Leung et al. 2002). Eggs are ubiquitously proclaimed potential propagules for the dispersal of invasive gobies, and we could empirically confirm this notion. In lieu of more conclusive evidence or quantitative knowledge on the relative importance of this vector, a preventive management should be installed based on previous experience with recreational boats as vectors for invertebrate species. For example, a preemptively installed “check–clean–dry” management measure of recreational boats originating from the goby source population would not only prevent the spread of goby propagules, but also of several other invasive species present in the local harbor such as the zebra and quagga mussels (*Dreissena* spp.) (Horvath 2008).

### Management implications

To install an effective preventive management, it is important that we adopt a holistic approach: not only accruing scientific knowledge on possible vectors, but also communicating with relevant experts that serve as decision makers about the feasibility of managing such vectors. After all, it is not the researchers that actually install the management. If decision makers do not consider a management measure feasible, they are unlikely to support the installation of such measures (Hirsch et al. 2015; N'Guyen et al. 2015). Recreational boats have long been assumed to be vectors and have been considered prime management targets in marine and freshwater systems (Johnson et al. 2001; Clarke Murray et al. 2011). Despite growing evidence for the relevance of recreational boats as vectors, their management has proven difficult to implement (but see, e.g., Horvath 2008). The barriers to implementation that we identified in our study are manifold. For a successful implementation of any management measure, the barriers identified by stakeholders should be appreciated and explored by researchers (Reed 2008). We argue that a solid scientific underpinning of the plausibility of a vector can serve as an important impulse for a transdisciplinary process toward a successful implementation. Further research on the measure should be designed in cooperation with experts outside academia to deliver relevant results improving the chances of management success (Reed 2008). Our ability to successfully prevent an imminent invasion is highest when we know least about the invasion: before ubiquitous propagule traffic allows for a scientific quantification of relative vector importance. Based on our empirical data and following the precautionary principle, it becomes clear that a preventive management of invasive gobies should consider eggs as propagules. For example, our study gives clues for when the pick up of propagules by recreational boats can occur and that existing boat drying measures need to be carefully re‐examined in light of the desiccation tolerance of invasive goby eggs.

## Conflict of Interest

The authors declare no conflict of interest.

## Supporting information


**Appendix S1.** Publications suggesting goby eggs as propagules and boats as vectors.

**Appendix S2.** Types of artificial substrates used by gobies for spawning in the harbor Basel.

**Appendix S3.** Map showing all 11 in‐stream barriers upstream of the potential source population and the numbers of passages of recreational boats across them.

**Appendix S4.** Detailed depiction of spawning traps used to retrieve eggs for experiments and to estimate the numbers of propagules spawned onto artificial substrates in the harbor where the potential source population has established.

**Appendix S5** ***.*** Information provided to the participants of the transdisciplinary workshop (transferred into English by the authors, square brackets: additional explanations to improve clarity for this paper).

**Appendix S6.** Questions provided in the questionnaire (transferred into English by the authors).

**Appendix S7.** Air exposure does not affect hatching dynamics of goby larvae.

**Appendix S8.** Questions provided in the questionnaire (transferred into English by the authors).

## References

[ece31892-bib-0001] Bonislavskaya, M. , A. Tanskii , A. Brisevich , A. O. Kozheletskaya , V. Vavzhinyak , and K. Formitskii . 2014 Peculiarities of embryonic development of round goby *Neogobius melanostomus* (Gobiidae) in fresh water. J. Ichthyol. 54:591–598.

[ece31892-bib-0002] Brenner, M. , and B. H. Buck . 2010 Attachment properties of blue mussel (*Mytilus edulis* L.) byssus threads on culture‐based artificial collector substrates. Aquacult. Eng. 42:128–139.

[ece31892-bib-0003] Castro, D. A. , and R. D. Podolsky . 2012 Holding On to a shifting substrate: plasticity of egg mass tethers and tethering forces in soft sediment for an intertidal gastropod. Biol. Bull. 223:300–311. 23264476 10.1086/BBLv223n3p300

[ece31892-bib-0004] Clarke Murray, C. , E. A. Pakhomov , and T. W. Therriault . 2011 Recreational boating: a large unregulated vector transporting marine invasive species. Divers. Distrib. 17:1161–1172.

[ece31892-bib-0005] Drake, D. A. R. , and N. E. Mandrak . 2014 Bycatch, bait, anglers, and roads: quantifying vector activity and propagule introduction risk across lake ecosystems. Ecol. Appl. 24:877–894. 24988783 10.1890/13-0541.1

[ece31892-bib-0501] Defila, R. , and A. Di Giulio . 2015 Integrating knowledge: challenges raised by the “Inventory of Synthesis”. Futures 65:123–135.

[ece31892-bib-0006] Fisk, J. M. , T. J. Kwak , R. J. Heise , and F. W. Sessions . 2013 Redd dewatering effects on hatching and larval survival of the robust redhorse. River Res. Appl. 29:574–581.

[ece31892-bib-0007] Gozlan, R. E. , D. Burnard , D. Andreou , and J. R. Britton . 2013 Understanding the threats posed by non‐native species: public vs. conservation managers. PLoS One 8:e53200. 23341931 10.1371/journal.pone.0053200 PMC3547005

[ece31892-bib-0008] Havel, J. E. , and J. B. Shurin . 2004 Mechanisms, effects, and scales of dispersal in freshwater zooplankton. Limnol. Oceanogr. 49:1229–1238.

[ece31892-bib-0009] Hirsch, P. E. , A. N'Guyen , I. Adrian‐Kalchhauser , and P. Burkhardt‐Holm . 2015 What do we really know about the impacts of one of the 100 worst invaders in Europe? A reality check. Ambio. doi:10.1007/s13280‐015‐0718‐9. 10.1007/s13280-015-0718-9 PMC4815762 26541873

[ece31892-bib-0010] Horvath, T. 2008 Economically viable strategy for prevention of invasive species introduction: case study of Otsego Lake, New York. Aquat. Invasions 3:3–9.

[ece31892-bib-0011] Incagnone, G. , F. Marrone , R. Barone , L. Robba , and L. Naselli‐Flores . 2015 How do freshwater organisms cross the “dry ocean”? A review on passive dispersal and colonization processes with a special focus on temporary ponds. Hydrobiologia 750:103–123.

[ece31892-bib-0012] Ishimatsu, A. , T. Takeda , Y. Tsuhako , T. T. Gonzales , and K. H. Khoo . 2009 Direct evidence for aerial egg deposition in the burrows of the Malaysian mudskipper, *Periophthalmodon schlosseri* . Ichthyol. Res. 56:417–420.

[ece31892-bib-0013] Johnson, L. E. , A. Ricciardi , and J. T. Carlton . 2001 Overland dispersal of aquatic invasive species: a risk assessment of transient recreational boating. Ecol. Appl. 11:1789–1799.

[ece31892-bib-0014] Kalchhauser, I. , P. Mutzner , P. E. Hirsch , and P. Burkhardt‐Holm . 2013 Arrival of round goby *Neogobius melanostomus* (Pallas, 1814) and bighead goby *Ponticola kessleri* (Günther, 1861) in the High Rhine (Switzerland). Bioinvasions Rec. 2:79–83.

[ece31892-bib-0015] Kornis, M. S. , N. Mercado‐Silva , and M. J. Vander Zanden . 2012 Twenty years of invasion: a review of round goby *Neogobius melanostomus* biology, spread and ecological implications. J. Fish Biol. 80:235–285. 22268429 10.1111/j.1095-8649.2011.03157.x

[ece31892-bib-0016] LaRue, E. A. , C. R. Ruetz III , M. B. Stacey , and R. A. Thum . 2011 Population genetic structure of the round goby in Lake Michigan: implications for dispersal of invasive species. Hydrobiologia 663:71–82.

[ece31892-bib-0017] Leprieur, F. , O. Beauchard , S. Blanchet , T. Oberdorff , and S. Brosse . 2008 Fish invasions in the world's river systems: when natural processes are blurred by human activities. PLoS Biol. 6:e28. 18254661 10.1371/journal.pbio.0060028 PMC2225436

[ece31892-bib-0018] Leung, B. , D. M. Lodge , D. Finnoff , J. F. Shogren , M. A. Lewis , and G. Lamberti . 2002 An ounce of prevention or a pound of cure: bioeconomic risk analysis of invasive species. Proc. Biol. Sci. 269:2407–2413. 12495482 10.1098/rspb.2002.2179 PMC1691180

[ece31892-bib-0019] Martin, K. L. , and A. L. Carter . 2013 Brave new propagules: terrestrial embryos in anamniotic eggs. Integr. Comp. Biol. 53:233–247. 23604618 10.1093/icb/ict018

[ece31892-bib-0020] N'Guyen, A. , P. E. Hirsch , I. Adrian‐Kalchhauser , and P. Burkhardt‐Holm . 2015 Improving invasive species management by integrating priorities and contributions of scientists and decision makers. Ambio. doi:10.1007/s13280‐015‐0723‐z. 10.1007/s13280-015-0723-z PMC4815759 26541874

[ece31892-bib-0021] Oulton, L. , P. Carbia , and C. Brown . 2013 Hatching success of rainbowfish eggs following exposure to air. Aust. J. Zool. 61:395–398.

[ece31892-bib-0022] Poos, M. , A. J. Dextrase , A. N. Schwalb , and J. D. Ackerman . 2010 Secondary invasion of the round goby into high diversity Great Lakes tributaries and species at risk hotspots: potential new concerns for endangered freshwater species. Biol. Invasions 12:1269–1284.

[ece31892-bib-0023] Preusse, O. 1924 Zur Fischverbreitungsfrage. Fischerei‐Zeitung 10:192–194.

[ece31892-bib-0024] Rahel, F. J. 2007 Biogeographic barriers, connectivity and homogenization of freshwater faunas: it's a small world after all. Freshw. Biol. 52:696–710.

[ece31892-bib-0025] Reed, M. S. 2008 Stakeholder participation for environmental management: a literature review. Biol. Conserv. 141:2417–2431.

[ece31892-bib-0026] Sakai, A. K. , F. W. Allendorf , J. S. Holt , D. M. Lodge , J. Molofsky , K. A. With , et al. 2001 Population biology of invasive species. Annu. Rev. Ecol. Syst. 32:305–332.

[ece31892-bib-0027] Schiemenz, F. 1925 Zur Widerstandsfähigkeit von Wildfischeiern gegen Lufttransport (Versuche an Stichlingseiern). Fischerei‐Zeitung 36:769–773.

[ece31892-bib-0028] Thacker, C. E. 2009 Phylogeny of gobioidei and placement within acanthomorpha, with a new classification and investigation of diversification and character evolution. Copeia 1:93–104.

[ece31892-bib-0029] Thienemann, A. 1950 Verbreitungsgeschichte der Süswassertierwelt Europas: versuch einer historischen Tiergeographie der europäischen Binnengewässer. Binnengewässer 18:156–159.

[ece31892-bib-0030] Tzankova, Z. , and A. Concilio . 2015 Controlling an invasive plant at the edge of its range: towards a broader understanding of management feasibility. Biol. Invasions 17:507–527.

[ece31892-bib-0031] Voigt, D. , and S. Gorb . 2010 Egg attachment of the asparagus beetle *Crioceris asparagi* to the crystalline waxy surface of *Asparagus officinalis* . Proc. Biol. Sci. 277:895–903. 19923132 10.1098/rspb.2009.1706 PMC2842725

[ece31892-bib-0032] Weissert, N. 2013 Recreational boating as an overland distribution vector for the invasive freshwater mussel Dreissena polymorpha in Switzerland. Master thesis. Swiss Federal Institute of Aquatic Science and Technology.

