# Corrigendum

**DOI:** 10.1002/ece3.2445

**Published:** 2016-09-09

The corrigendum is for the article: Hirsch PE, Adrian‐Kalchhauser I, Flämig S, N'Guyen A, Defila R, Di Giulio A, Burkhardt‐Holm P (2016). A tough egg to crack: recreational boats as vectors for invasive goby eggs and transdisciplinary management approaches. *Ecology and Evolution* **6**: 707–715 “ DOI – 10.1002/ece3.1892.

In the caption to Figure 2, the first mentioning of the year number 2013 should be corrected to 2014.

So the text “Number of goby eggs spawned on artificial substrates by the potential source population in 2013” should read “Number of goby eggs spawned on artificial substrates by the potential source population in 2014.”

Thank you very much in advance for your assistance!

