# Supplementary material for: A tough egg to crack: recreational boats as vectors for invasive goby eggs and transdisciplinary management approaches
Source: Ecol Evol. 2016 Jan 11;6(3):707–15. doi: 10.1002/ece3.1892 (PMC4739576; doi:10.1002/ece3.1892)
Supplement: Supplementary file 1 — Appendix S1. Publications suggesting goby eggs as propagules and boats as vectors. [file ECE3-6-707-s001.docx]

Appendix S1: **Publications suggesting goby eggs as propagules and boats as vectors**

Ahnelt, Harald; Banarescu, Petru; Spolwind, Robert; Harka, Akos; Waidbacher, Herwig (1998): Occurrence and distribution of three gobiid species (Pisces, Gobiidae) in the middle and upper Danube region - examples of different dispersal patterns? Biologia 53 (5).

Hensler, Stephen R.; Jude, David J. (2007): Diel vertical migration of round goby larvae in the great lakes. Journal of Great Lakes Research 33 (2), pp. 295–302.

Jude, D. J.; Janssen, J.; Crawford, G. (1995): Ecology, distribution, and impact of the newly introduced round tubenose gobies on the biota of the St Clair and Detroit Rivers. In D. A. Wilcox (Ed.): The role of wetlands as nearshore habitat in Lake Huron (Ecovision World Monograph Series), pp. 447–460.

Moskal'kova, K. I. (1996): Ecological and morphophysiological prerequisites to range extention in the round goby *Neogobius melanostomus* under conditions of anthropogenic pollution. Journal of Ichtyology 36 (8), pp. 584–590.

Ray, W. J., & Corkum, L. D. 2001. Habitat and site affinity of the round goby. Journal of Great Lakes Research, 27(3), 329-334.

Sokolov, L. I.; Sokolova, E. L.; Pegasov, V. A.; Shatunovskii, M. I.; Kistenev, A. N. (1994): Ichthyofauna of the Moskva River within the city of Moscow: Some data on the state of the ichthyofauna. Vopsy Ikhtiologii 34 (5), pp. 634–641.

Tsepkin, E.A; Sokolov, L. I.; Rusalimchik (1992): Ecology of the round goby *Neogobius melanostomus*, an occasional colonizer of the basin of the Moskva river. Biologiceskie nauki, pp. 46–51.
