# Supplementary material for: A tough egg to crack: recreational boats as vectors for invasive goby eggs and transdisciplinary management approaches
Source: Ecol Evol. 2016 Jan 11;6(3):707–15. doi: 10.1002/ece3.1892 (PMC4739576; doi:10.1002/ece3.1892)
Supplement: Supplementary file 2 — Appendix S2. Types of artificial substrates used by gobies for spawning in the harbor Basel. [file ECE3-6-707-s002.docx]

*
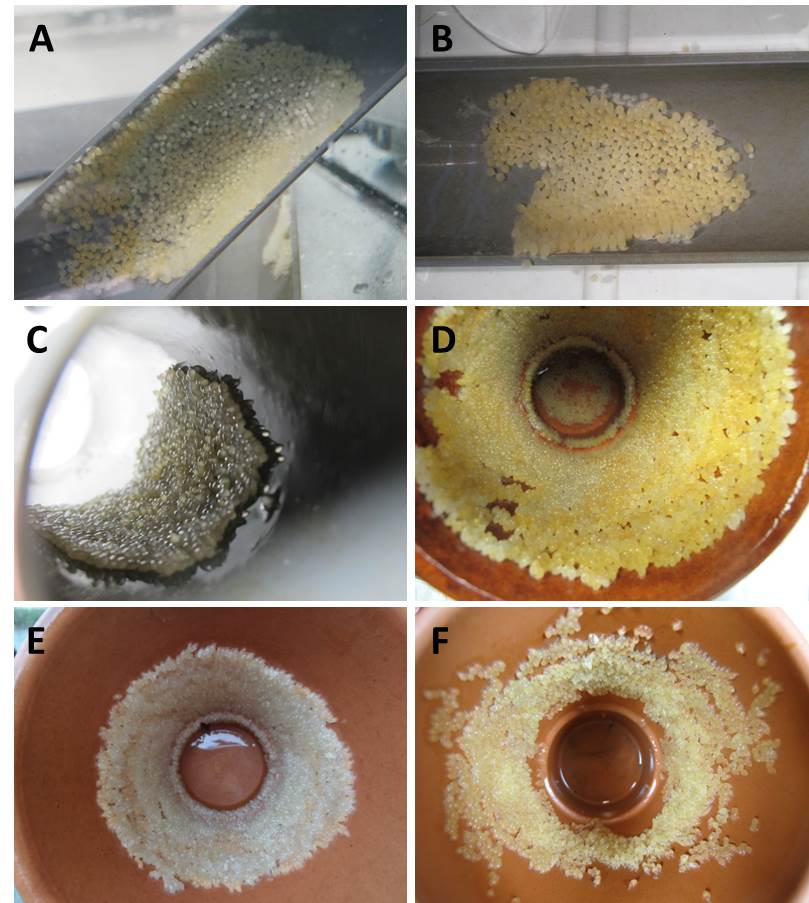
*

Appendix S2: **Types of artificial substrates used by gobies for spawning in the harbor Basel**. Gobies lay their eggs on PVC tubes (A, B, C) and clay pots (D, E, F).
