# Supplementary material for: A tough egg to crack: recreational boats as vectors for invasive goby eggs and transdisciplinary management approaches
Source: Ecol Evol. 2016 Jan 11;6(3):707–15. doi: 10.1002/ece3.1892 (PMC4739576; doi:10.1002/ece3.1892)
Supplement: Supplementary file 3 — Appendix S3. Map showing all 11 in‐stream barriers upstream of the potential source population and the numbers of passages of recreational boats across them. [file ECE3-6-707-s003.docx]

*
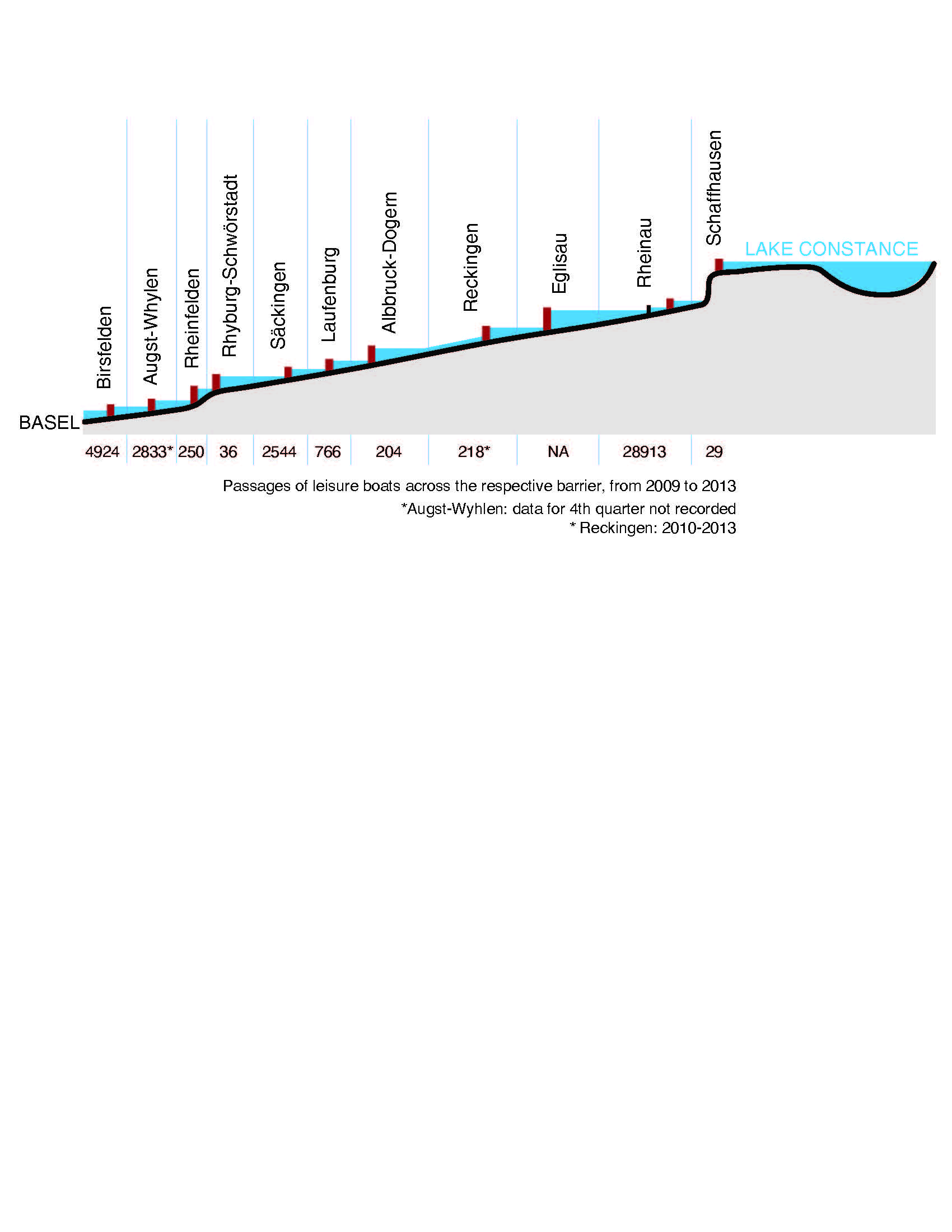
*

Appendix S3: **Map showing all 11 in-stream barriers upstream of the potential source population and the numbers of passages of recreational boats across them.**

We first mapped the upstream migration route from the harbor in Basel where gobies first appeared to the next large alpine lake: Lake Constance. Lake Constance is free of gobies and socio-economically important due to its attraction to tourists and commercial fisheries. In total, there are 11 in-stream barriers which all are power plants, except for Schaffhausen, which is a natural waterfall. Transports of boats across these barriers are managed as a public service and the power plant operators also protocol this service. To gather data on boat passages for the years 2009 to 2013, a questionnaire (one for each year) was sent to staff organizing upstream transfer of boats across in-stream barriers. The questionnaire is available upon request. Only boat transports that use the available facilities are recorded. Smaller boats such as canoes or rubber boats are usually not included.
