# Supplementary material for: A tough egg to crack: recreational boats as vectors for invasive goby eggs and transdisciplinary management approaches
Source: Ecol Evol. 2016 Jan 11;6(3):707–15. doi: 10.1002/ece3.1892 (PMC4739576; doi:10.1002/ece3.1892)
Supplement: Supplementary file 4 — Appendix S4. Detailed depiction of spawning traps used to retrieve eggs for experiments and to estimate the numbers of propagules spawned onto artificial substrates in the harbor where the potential source population has established. [file ECE3-6-707-s004.docx]

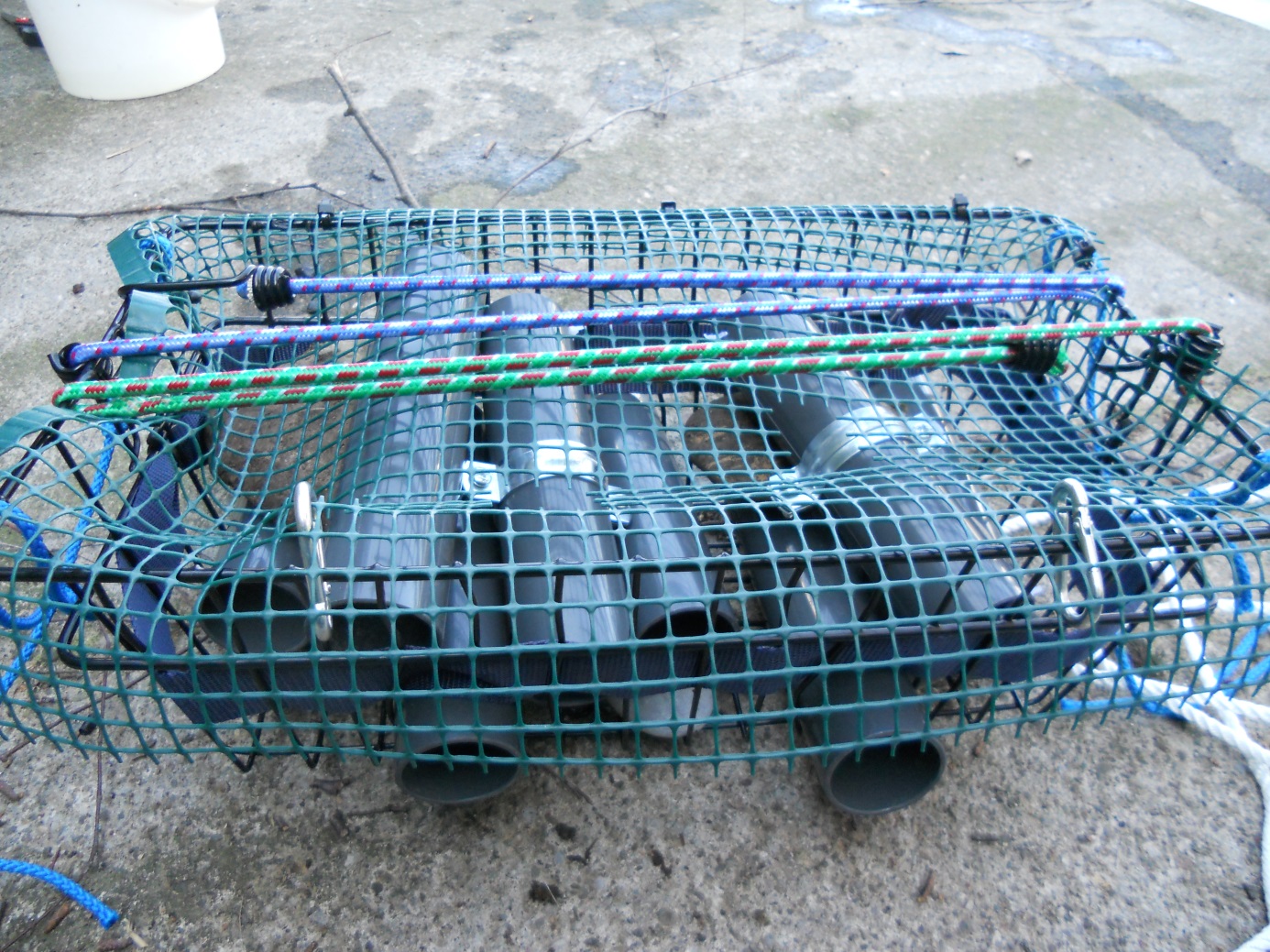


Appendix S4: **Detailed depiction of spawning traps used to retrieve eggs for experiments and to estimate the numbers of propagules spawned onto artificial substrates in the harbor where the potential source population has established.**

Spawning traps consisted of the following items: a pannier filled with several ~10x5 cm stones as ballast weight, four standard clay pots (top diameter: 10.5 cm, bottom diameter: 5.5 cm, height: 13 cm), and seven grey PVC tubes (diameter: 4.5 cm, length: 20 cm). Five spawning traps were lowered to the harbour basin bottom at approximately 4 m depth and retrieved and checked for eggs once a week. Sampling commenced on the27^th^ of February and was continued as long as eggs were retrieved. In October, after no eggs were retrieved any more for two weeks we stopped sampling.
