# Supplementary material for: A tough egg to crack: recreational boats as vectors for invasive goby eggs and transdisciplinary management approaches
Source: Ecol Evol. 2016 Jan 11;6(3):707–15. doi: 10.1002/ece3.1892 (PMC4739576; doi:10.1002/ece3.1892)
Supplement: Supplementary file 5 — Appendix S5 . Information provided to the participants of the transdisciplinary workshop (transferred into English by the authors, square brackets: additional explanations to improve clarity for this paper). [file ECE3-6-707-s005.docx]

Appendix S5*.* **Information provided to the participants of the transdisciplinary workshop (transferred into English by the authors, square brackets: additional explanations to improve clarity for this paper)**

*Recommendation for management: Inspecting and cleaning of boats*

A mandatory control, cleaning and drying ("check, clean, dry") of recreational boat hulls prior to transfer into another water body or another section of a water body will be implemented. This will take place together with boating clubs, organizers of water sport competitions etc. so that it becomes a broadly accepted measure.

*Expected effect:*

In the ideal case, this management can preclude a further spread of gobies in the form of eggs attached to recreational boats. In any case, however, will the propagule pressure be strongly reduced. A lower propagule pressure will make a further spread less likely and reduce the population growth of newly introduced populations.

*Possible scenario in case the management is not implemented:*

Following the translocation of recreational boats, gobies will be spread across Switzerland following a pattern that will be hard to predict. Possibly implemented measures to halt the spread at in-stream barriers [such as e.g. impassable fish ladders] will be ineffective because these will be overcome by boats that are translocated across such barriers.

A monitoring focussing on current invasion hot-spots in Switzerland will fall short of its desired effect of early detection because the spread of gobies will be saltatorial and not continuous. Fishers, freshwater ecosystem managers and interested groups do not expect such a sudden appearance of gobies [which occurrs because of the saltatorial spread]. They are unprepared and respond late or not at all to a detection of gobies which suddenly appear as a result of human aided long-distance dispersal.

*Basis for recommendation of management:*

Observations in the field indicate that gobies use boat hulls in shallow shoreline areas as spawning substrate. Trials using spawning traps have revealed that gobies accept artificial substrates for spawning. Boat hulls constitute such artifical surfaces.
