# Supplementary material for: A tough egg to crack: recreational boats as vectors for invasive goby eggs and transdisciplinary management approaches
Source: Ecol Evol. 2016 Jan 11;6(3):707–15. doi: 10.1002/ece3.1892 (PMC4739576; doi:10.1002/ece3.1892)
Supplement: Supplementary file 6 — Appendix S6. Questions provided in the questionnaire (transferred into English by the authors). [file ECE3-6-707-s006.docx]

Appendix S6**: Questions provided in the questionnaire (transferred into English by the authors)**

1. Based on your knowledge and with respect to containing the potential source population or preventing its further spread, how effective do you consider the proposed management measure?

2. How urgent do you personally consider the implementation of the proposed measure?

3. How straightforward is the implementation, do you see hardly any or major barriers?

4. Do you have further comments concerning the proposed management measure or concerning your answers?
