# Supplementary material for: A tough egg to crack: recreational boats as vectors for invasive goby eggs and transdisciplinary management approaches
Source: Ecol Evol. 2016 Jan 11;6(3):707–15. doi: 10.1002/ece3.1892 (PMC4739576; doi:10.1002/ece3.1892)
Supplement: Supplementary file 7 — Appendix S7. Air exposure does not affect hatching dynamics of goby larvae. [file ECE3-6-707-s007.docx]

*
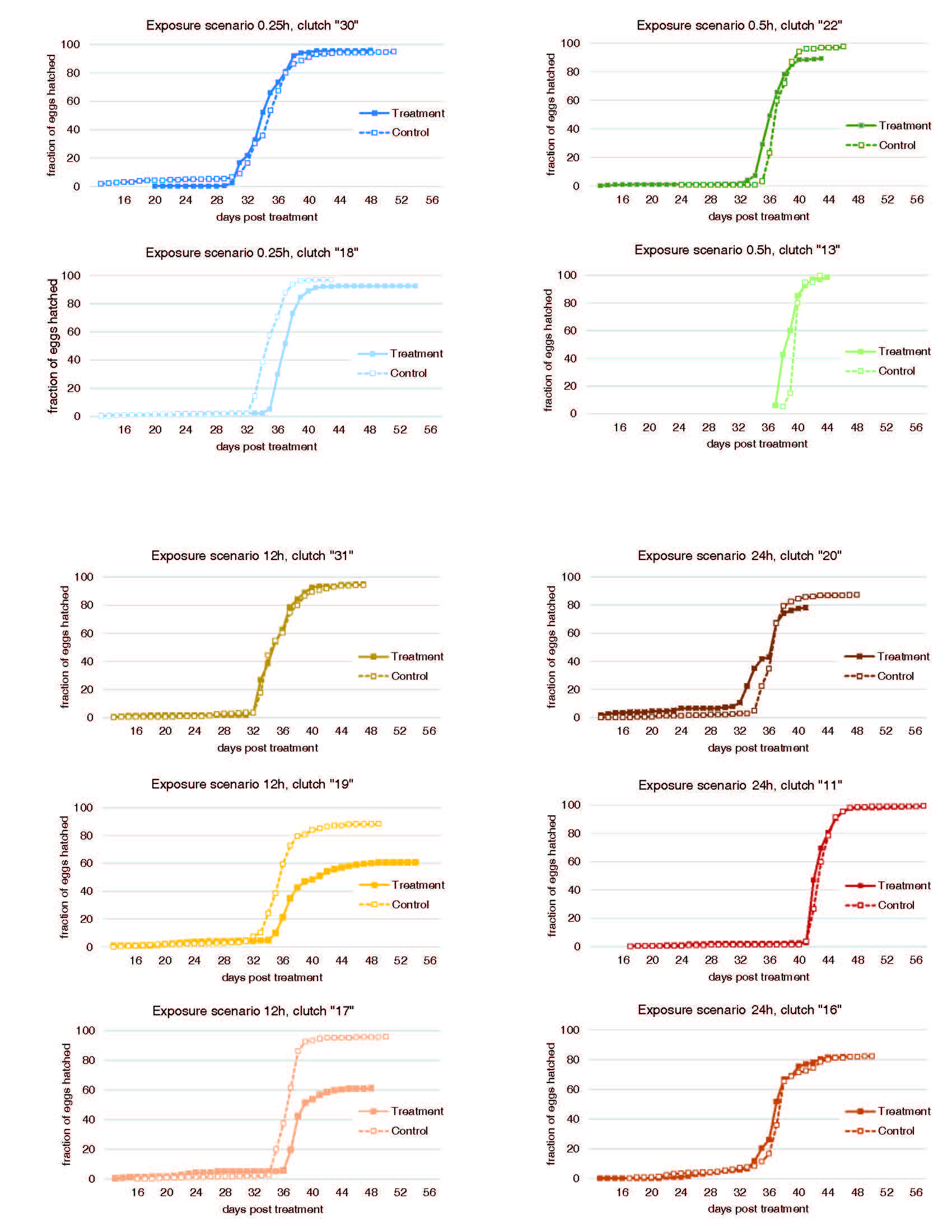
*

Appendix S7: **Air exposure does not affect hatching dynamics of goby larvae.** Cumulative hatching curves from all 10 clutches used for the air-exposure experiment. For cumulative hatching success, daily hatching success rates were summed up. Clutch numbers are for internal purposes.
