# Supplementary material for: A tough egg to crack: recreational boats as vectors for invasive goby eggs and transdisciplinary management approaches
Source: Ecol Evol. 2016 Jan 11;6(3):707–15. doi: 10.1002/ece3.1892 (PMC4739576; doi:10.1002/ece3.1892)
Supplement: Supplementary file 8 — Appendix S8. Questions provided in the questionnaire (transferred into English by the authors). [file ECE3-6-707-s008.docx]

Appendix S8. **Workshop participants’ comments provided in written form (transferred into English by the authors, square brackets: additional explanations to improve clarity for this paper).**

Civil Society 1

CivSoc1.5: Reasonable also against the spread of diseases and other invasive species.

CivSoc1.6: Because this management is very sophisticated, it would only be acceptable if it is proven that gobies spread via this vector in large numbers.

Civil Society 2

CivSoc2.1: I could imagine that a control of "check, clean and dry" would be difficult.

CivSoc2.2: Efficiency: on the borderline to being effective. Barriers to implementation: enforcement and control. Important is awareness raising ++ = self-responsibility to act.

CivSoc2.3: As mentioned during the first meeting [refers to the first stakeholder workshop of our project] it is very difficult to enforce this cleaning. There are an estimated 10,000 boats per year.

Scholars

Sc.1: By all means necessary, especially in concert with the monitoring [another recommended management option]. Presumably difficult to implement and costly. In the case of Basel: rather few boats, therefore less of an effect?

Sc.2: Efficiency not yet completely clear.

Sc.3: Efficiency depends on whether this is a relevant form of spread.

Authorities/administration

Adm.1: Depends on whether this is a primary vector. Is the control of commercial ships not an issue any more?

Adm.2: We already performed a boat inspection. It became obvious that specific parts are hardly accessible and hence hardly cleanable.

Adm.3: Barriers to implementation: there probably is a complete lack of any legislative basis. Advantage: would also counteract the spread of other organisms.

Adm.4: Difficult to implement! New administrative regulations are always troublesome!

Adm.5: Enforcement requires adequate resources – are these available?
